# Pharmacological Myopia Control Influence on Quality of Life and Psyche among Adolescents

**DOI:** 10.3390/jcm9123920

**Published:** 2020-12-03

**Authors:** Andrzej Michalski, Małgorzata Rogaczewska, Magdalena Maleszka-Kurpiel, Marcin Stopa

**Affiliations:** 1 Department of Ophthalmology, Chair of Ophthalmology and Optometry, Poznan University of Medical Sciences, 60-780 Poznan, Poland; malgorzata.rogaczewska@gmail.com (M.R.); stopa@ump.edu.pl (M.S.); 2 Optegra Eye Health Care Clinic in Poznan, 61-101 Poznan, Poland; m.maleszka-kurpiel@optegra.com.pl; 3 Department of Optometry, Chair of Ophthalmology and Optometry, Poznan University of Medical Sciences, 60-780 Poznan, Poland

**Keywords:** myopia control, low dose atropine, self-esteem, psyche in myopia control

## Abstract

Myopia is a global problem affecting all aspects of patients’ lives. Objectives: The aim of the study was to evaluate the influence of low dose atropine (LDA) myopia control on the quality of life in patients with myopia. Material and Methods: A self-constructed questionnaire, including eight questions, was distributed among 40 patients. The questionnaire was divided into two subsections: (1) influence of LDA on visual functions and (2) influence of LDA on self-esteem. Answers were collected separately for boys (18 patients) and girls (22 patients) and compared considering spherical equivalent (SE) and myopia progression rate. Results: Girls reported more issues with near activities and pupil size. Boys and girls complained similarly, regarding the sun glare. We found a high level of certainty about the efficacy of LDA therapy among both examined groups and a little improvement in self-esteem. Girls recommended LDA therapy more often than boys, especially when the progression rate was low. There was no statistically significant difference in answer scores between groups with different myopia progression rates for boys. Girls with lower progression rates reported more issues with near work and sun glare and less trust in LDA therapy’s effectiveness than girls with a higher progression rate. There was no statistically significant correlation between SE and the total answer score for both genders. Conclusions: Findings concerning childrens’ and adolescents’ psyche are a new aspect of myopia control. We prove that patients during pharmacological myopia control did not report significant problems caused by LDA therapy and they were convinced about its efficacy, had greater self-esteem, and recommended it to peers.

## 1. Introduction

The increasing prevalence of myopia is a significant medical and social problem worldwide. It is estimated that in 2030 more than 3.3 billion people will be myopic, and in 2050, even up to 4.76 billion. Nowadays, high myopia is observed in 5.2% of the global population and it will increase to 9.8% by 2050 [1]. According to the literature, myopic refractive error is related to a higher risk of ocular pathology, particularly retinal detachment [2,3]. For a spherical equivalent from −1.0 to −3.0 diopters, the risk of rhegmatogenous retinal detachment increases fourfold compared with an emmetropic eye, and if myopia is greater than −3.0 diopters, the risk increases tenfold [2]. In addition to ocular complications, myopia is a well-known factor that compromises affected individuals’ daily lives. Rose et al. qualitatively analyzed the quality of life (QoL) of patients with myopia. They reported that patients, notably, with high myopia, gave more examples of limitations in everyday life. Their lack of self-confidence and social handicap resulted in a lower quality of life [4].

The Quality of Life Impact of Refractive Correction (QIRC) is useful to detect differences in quality of life impact from various refractive corrections (spectacles, contact lenses, and refractive surgery). Pesudovs et al. reported that the QIRC score was highest in refractive surgery patients (mean QIRC score of 50.2 ± 6.3), followed by contact lens wearers (mean QIRC score of 46.7 ± 5.5) and spectacle wearers (mean QIRC score of 44.1 ± 5.9) [5]. Additionally, Ghee et al. and Tounka et al. established that surgical methods of myopia treatment improved quality of life of operated patients [6,7]. Many methods for slowing myopia progression are currently in use, such as topical low-dose atropine, soft bifocal or multifocal contact lenses (also experimental designs), and orthokeratology contact lenses [8,9,10,11,12,13]. Additionally, combined therapies are recommended, e.g., low-dose atropine and orthokeratology [14].

Atropine (0.01%, 0.025%, 0.05%, 0.1%, 0.5%, and 1%) has been used to control myopia progression [8,9,10,11]. Atropine affects pupil size and accommodation amplitude. The low-dose atropine (0.01%), used in myopia treatment, has minimal effect on pupil size, accommodation, and near vision [8]. Patients using atropine therapy may experience blurred vision, glare, or photophobia. Atropine also causes allergic reactions [15]. In the recent findings, 0.05% atropine seems to be most effective in myopia control [10].

Adolescence is a critical developmental period. Teenagers are adjusting to physical changes, looking for personal identity and independence, exploring their sexuality. They are also relying more and more on friendship groups [16].

It has to be emphasized that exploring patients’ satisfaction with therapy and their quality of life should be essential in the medical care process. Fitzpatrick stated that patient satisfaction is an important outcome measure [17]. Moreover, a positive correlation between satisfaction and compliance is proven [18]. The latter aspect is particularly vital in low dose atropine (LDA) treatment because it is a long-term procedure [8,10], therefore based on the patient’s and his/her family’s compliance.

Another aspect of questionnaire examinations is the message, that not only objective results of the treatment should be important for medical staff, but also the healed person’s statements and psyche. This could be another way to empower patients in the treatment process. We aimed to check selected factors of life of myopic patients because the ophthalmic quality of life is connected with various aspects, e.g., activity limitation, visual and ocular-comfort symptoms, and general health concerns. It also influences emotional, social, and economic issues [19]. Data published by Katz et al. showed that near-sighted children had lower self-esteem and were lonelier. They were also more criticized for their physical attributes. The authors’ findings also proved that myopic children experienced more fear and stressful events during childhood. Those results underlined the relation between psychological stress, emotions, and myopic refractive error in children [20]. As Łazarczyk et al. showed, nearsightedness increases the level of trait anxiety in 13–14-year-olds, especially in boys. The authors also suggested the possibility of worsened bullying against myopes, resulting in greater anxiety and depression disorders. These problems validate the careful analysis of psychological problems in young myopes, especially in boys [21].

## 2. Experimental Section

### 2.1. Aim

The aim of this study was to evaluate the influence of LDA on the quality of life in patients with myopia. The clinical symptoms (photophobia and difficulties with near work) and psyche of adolescent patients, regarding their functioning in the peer group, were assessed.

### 2.2. Material and Methods

In total, 18 male participants (median age: 12.5 years; range: 9–15 years) and 22 female participants (median age: 13.5 years; range: 10–16 years) were examined.

A self-constructed questionnaire, including 8 questions, was divided into 2 subsections: (1) influence of LDA on visual functions—questions 1–4; (2) influence of LDA on self-esteem—questions 5–8 (Supplementary S1). Each question was rated between 1–10 points. The lower the result, the lower the negative influence of LDA therapy on visual function and better self-esteem (1 for no problems/most satisfied, 10 for many problems and no satisfaction from treatment). A questionnaire was given to the patients after 1 year of 0.01% atropine application for myopia control. All visits included full ophthalmological examination with cycloplegic refraction performed after the instillation of tropicamide 1% three times (Tropicamidum WZF 1%, Polfa S.A., Warsaw, Poland), with an auto kerato-refractometer measurement (Topcon KR 8900, Tokyo, Japan). The axial length of the eyes was measured with an optical biometer (Zeiss IOL Master 4, Jena, Germany). Best-corrected visual acuity (BCVA) was assessed with the Snellen chart and converted to logMAR units. Only patients with BCVA 0.00 or better were included in the study. The spherical equivalent (SE) for each patient was calculated as the mean of values for the right and left eyes.

The exclusion criterion was any ophthalmic or systemic disease.

A detailed explanation of the myopia treatment regimen was provided prior to the therapy’s onset and repeated during subsequent visits. The questionnaire was introduced before the ophthalmological examination at the control visits after 1 year of therapy. The question list was concisely explained to the patients and their parents, and they were also assured that the obtained data would be published anonymously. Each participant was asked afterward if he/she understood the phrase “progression of refractive error”. Participants were given up to 10 min to complete the questionnaire. Their parents were asked not to interfere in any way with their child during this period.

The study was conducted in accordance with the Declaration of Helsinki and approved by the Ethics Committee of Poznan University of Medical Sciences (project identification code 1042/19), and a written informed consent was obtained from one parent of the examined individual.

The Shapiro–Wilk test was used to determine the normality of continuous variables. All data were not normally distributed, thus are presented as median and minimum–maximum values. Mean values ± standard deviation (SD) were provided for informative purposes. The Mann–Whitney U-test with continuity correction was used to compare the differences between variables. Spearman correlations were calculated for selected data. A *p*-value of less than 0.05 was considered significant. All statistical analyses were performed with Statistica 13.3 (TIBCO Software Inc., Palo Alto, CA, USA).

## 3. Results

The BCVA of all tested eyes was 0.00 logMAR or better. Median SE for boys was −2.88 (−12.25; −1.50) D and for girls −3.12 (−8.5; −1.25) D.

The answer scores for boys and girls are presented in Table 1. Answer scores for both genders considering myopia progression rate are summarized in Table 2; the Spearman correlation between SE and total answer scores for boys (r = −0.98; *p* = 0.696) and girls (r = 0.257; *p* = 0.248).

## 4. Discussion

We focused on selected possible side effects of LDA therapy and patients’ psyche. Our results concerning clinical symptoms are similar to other authors’ findings. In both male and female subjects, some issues were reported.

Girls reported more issues with near activities. However, it has to be noted that median values were equal for the first and second questions of the questionnaire (meaning: I never have issues) for both groups. Furthermore, in examined groups, only a few times, significant problems with near work were reported. Cooper et al. concluded that the maximal atropine dose, which can be applied without clinical symptoms, was 0.02% [22]. Loughman and al. found no statistically significant receding of near-point convergence and no reduced accommodation amplitude when using 0.01% atropine in students [23]. Chia et al. reported the loss of accommodation (2−3 D), which is a minimal and significant influence of 0.01% atropine use on near visual acuity [8]. In results published by Yam et al., loss of accommodation amplitude is in the range of 0.5 D, and there is no significant reduction of near visual acuity [9,10].

There was no statistically significant difference between boys and girls regarding the answer for sun glare. In the Atropine for the Treatment of Myopia 1 (ATOM1) study using 1% atropine, some children quit the study because of glare issues, but there were no serious issues when using atropine 0.01% during the ATOM2 study [8,11]. It has to be noted that girls complained more about pupil size, suggesting that appearance was more critical for them and/or their peers than for boys. Pupil size increases significantly during 0.01% atropine use [23], but the increase is about 1 mm and lower than when using other atropine doses [8]. The Low-Concentration Atropine for Myopia Progression (LAMP) study reported a change in pupil size that was even smaller, in the range of 0.5 mm [9,10].

There are no published studies on the psychological aspect of LDA therapy; therefore, direct comparison of our results with others is impossible. The patients’ age range was chosen deliberately because self-esteem and interactions with peers are crucial for adolescents. The interaction with a peer group influences health-related behavior in both positive and risky ways [16]. A high level of certainty about the efficacy of LDA therapy among both examined groups could be a positive factor in improving compliance. Kincey et al. stated that compliance was significantly correlated to satisfaction score [18]. No statistically significant differences between groups for questions six and seven were found. It has to be noted that median score ranges were [1,2,3,4,5] for both groups, meaning a positive influence or no influence. Mean scores for both questions and both genders were close to four, which meant “self-esteem improved”.

Myopic patients are reported as having to deal with some psychological issues. Myopes are mentally closed and tend to be very conscientious [24]. Additionally, Beedle et al. reported that myopes are introverted [25]. On the contrary, Berg et al. found no specific myopic personality [26]. Aspects of psychoanalysis in myopic patients published by Seitler underlined issues with separation fears and castration anxiety [27]. Regarding this and our findings, each improvement of myopic child psyche is essential. This is a newly described and positive aspect of LDA therapy.

Girls recommend LDA therapy more often than boys, especially when the progression rate is low. Talking about the refractive error and its ongoing therapy may improve adolescents’ position among friends.

For boys, there is no statistically significant difference in answer scores between groups with progression rates ≥−0.25 D/year and −0.50/year. Interestingly, girls with lower progression rates reported more issues with near work and sun glare and less trust in LDA therapy’s effectiveness than girls with a higher progression rate. It has to be noted that girls with lower progression rates recommend LDA therapy to peers more often despite the complaints.

When analyzing inter-gender differences, we found that girls complained more often about near work issues and sun glare than boys in the lower progression rate group, although they recommended LDA therapy to peers. For a higher progression rate, we did not find statistically significant differences.

There is no statistically significant correlation between SE and the total answer score for both genders. However, our results suggest that boys are less concerned about LDA therapy when SE is less myopic and girls tend to have the opposite reaction. These results suggest that boys are more concerned about refractive error value, and when myopia is low, they do not complain. Conversely, girls accept eventual LDA side effects when the refractive error is high and in other situations, they tend to complain more.

Our results proved that:

- LDA therapy causes no profound subjectively-reported side effects;
- For teenagers, having the possibility of slowing down the refractive error progression is especially important. Both boys and girls trust the eye care specialist who provides LDA therapy. Both boys and girls have a little bit higher self-esteem. They also both recommend LDA therapy to peers—especially girls—significantly more often than boys and often when the progression rate is low.

Findings concerning childrens’ and adolescents’ psyche are a new aspect of myopia control. We want to emphasize that patients during myopia control do not have significant problems caused by LDA therapy and they are convinced about its efficacy and recommend it to peers.

## 5. Conclusions

Due to the rapid increase in the incidence of myopia and atropine treatment, it becomes essential to determine its impact and the effects of treatment on the quality of life of adolescents.

Our study showed that LDA myopia control over an extended period is well tolerated regarding subjective clinical symptoms. It has to be emphasized that this pharmacological procedure had a positive impact on patients’ psychological state. They trusted the ophthalmologist who provided this therapy and recommended it. LDA therapy also had a little positive impact on self-esteem.

The positive impact of LDA on the patient’s psyche is another crucial factor that should favor the early initiation of pharmacological myopia control in individuals with nearsightedness progression.

Limitation of the study: small sample. Regarding this, we were unable to perform detailed statistical analysis for low, moderate, and high myopia subgroups. Additionally, the clinical significance of our results needs to be further investigated.

What was known: LDA influence on visual functions.

What this study adds: Pharmacological myopia control improves patients’ psyche.

## Figures and Tables

**Table 1:** Answer scores for both groups presented as the median value (range) and *p* (Mann−Whitney U-test with continuity correction); scores for each question for both groups presented as mean value ± SD.

Question	Score: Boys	Score: Girls	*p*	Score: Boys	Score: Girls
1	1 (1–7)	1 (1–10)	0.047	1.39 ± 1.42	2.14 ± 2.17
2	1 (1–2)	2 (1–6)	0.001	1.06 ± 0.24	2.55 ± 1.90
3	3 (1–7)	2 (1–10)	0.461	2.94 ± 1.51	3.18 ± 2.68
4	1 (1–9)	2.5 (1–8)	0.045	2.17 ± 2.31	3.95 ± 3.17
5	1 (1–4)	1 (1–4)	0.413	1.44 ± 0.85	1.64 ± 0.90
6	5 (1–5)	5 (1–5)	0.540	4.06 ± 1.47	4.18 ± 1.40
7	4.5 (1–5)	5 (1–5)	0.331	4.06 ± 1.30	4.32 ± 1.21
8	5 (1–5)	3 (1–5)	0.003	4.11 ± 1.23	2.91 ± 1.15

**Table 2:** Answer scores for: groups of boys with progression ≥−0.25 D/year (*n* = 16) and −0.50 D (*n* = 2) and groups of girls with progression ≥−0.25 D/year (*n* = 14) and −0.50 D (*n* = 8), presented as median value (range) and *p* (Mann−Whitney U-test with continuity correction). In the last two columns, *p* for inter-gender differences in scores for both myopia progression rates is presented.

Question	Score: Boys Progression ≥−0.25 D/Year	Score: Boys Progression −0.50 D/Year	*p*	Score: Girls Progression ≥−0.25 D/Year	Score: Girls Progression −0.50 D/Year	*p*	Boys vs. Girls Progression ≥−0.25 D/Year *p*	Boys vs. Girls Progression −0.50 D/Year *p*
1	1 (1–7)	1 (1–1)	0.700	2 (1–10)	1 (1–2)	0.038	0.015	0.803
2	1 (1–2)	1 (1–1)	0.860	2 (1–6)	1.5 (1–6)	0.971	0.002	0.303
3	3 (1–7)	2 (2–2)	0.206	3.5 (1–10)	1 (1–4)	0.004	0.496	0.076
4	1 (1–9)	1.5 (1–2)	0.790	4.5 (1–8)	1 (1–8)	0.195	0.019	1
5	1 (1–3)	2.5 (1–4)	0.327	2 (1–4)	1 (1–2)	0.041	0.058	0.263
6	4.5 (1–5)	5 (5–5)	0.240	5 (2–5)	5 (1–5)	0.409	0.246	0.42
7	5 (1–5)	3 (3–3)	0.080	5 (3–5)	4.5 (1–5)	0.107	0.211	0.684
8	4.5 (1–5)	5 (5–5)	0.245	2.5 (1–5)	4 (1–5)	0.072	0.004	0.075

## Supplementary Materials

The following are available online at https://www.mdpi.com/2077-0383/9/12/3920/s1, supplementary S1: Questionnaire.
