# Supplementary material for: Pharmacological Myopia Control Influence on Quality of Life and Psyche among Adolescents
Source: J Clin Med. 2020 Dec 3;9(12):3920. doi: 10.3390/jcm9123920 (PMC7761740; doi:10.3390/jcm9123920)
Supplement: Supplementary file 1 [file jcm-09-03920-s001.pdf]

Please answer the questions marking one number in range 1-10 with a circle:

1. During the day (especially in the morning), do you experience any problems with up-close activities?

1 2 3 4 5 6 7 8 9 10

never very rarely rarely sometimes often very often

2. Do you take your glasses off for close-up activities (or you do not want to wear contact lenses)?

1 2 3 4 5 6 7 8 9 10

never very rarely rarely sometimes often very often

3. Do you feel glare by sunlight / other types of light?

1 2 3 4 5 6 7 8 9 10

never very rarely rarely sometimes often very often

4. Has anyone (parents / siblings / peers) pointed out to you that your pupils are wider?

1 2 3 4 5 6 7 8 9 10

never very rarely rarely sometimes often very often

5. Do you know that using prescribed eyedrops, progression of your refractive error will be slower?

1 2 3 4 5 6 7 8 9 10

I am sure rather yes I have no opinion I have doubts It does not work

6. From the beginning of using the eyedrops, your self-esteem is:

1 2 3 4 5 6 7 8 9 10

much higher higher same rather lower lower

7. From the beginning of using the eyedrops do you feel more confident in your class/among peers?

1 2 3 4 5 6 7 8 9 10

yes - much more confident /a little more confident / same / I feel less confident I feel bad

8. Have you passed the information about the possibility of slowing down the progression of the refractive error to your peers with a similar refractive error (myopia - "minuses"?).

1 2 3 4 5 6 7 8 9 10

Yes, to many / only a few I am not talking about it / I am talking about doubts / I discourage others
